# Genome Sequencing of West Nile Virus from Human Cases in Greece, 2012

**DOI:** 10.3390/v5092311

**Published:** 2013-09-24

**Authors:** Luisa Barzon, Anna Papa, Monia Pacenti, Elisa Franchin, Enrico Lavezzo, Laura Squarzon, Giulia Masi, Thomas Martello, Theodolinta Testa, Riccardo Cusinato, Giorgio Palù

**Affiliations:** 1 Department of Molecular Medicine, University of Padova, 35122 Padova, Italy; E-Mails: luisa.barzon@unipd.it (L.B.); elisa.franchin@unipd.it (E.F.); enrico.lavezzo@unipd.it (E.L.); laura.squarzon@unipd.it (L.S.); giulia.masi@unipd.it (G.M.); thomas.martello@unipd.it (T.M.); 2 Microbiology and Virology Unit, Padova University Hospital, 35122 Padova, Italy; E-Mails: monia.pacenti@sanita.padova.it (M.P.); riccardo.cusinato@sanita.padova.it (R.C.); 3 Department of Microbiology, Medical School, Aristotle University of Thessaloniki, Thessaloniki 54621, Greece; E-Mails: annap@med.auth.gr (A.P.); thtesta@auth.gr (T.T.)

**Keywords:** West Nile virus, genome sequencing, phylogenetic analysis, Greece, human surveillance, neuroinvasive disease

## Abstract

A West Nile Virus (WNV) lineage 2 strain, named Nea Santa-Greece-2010, has been demonstrated to be responsible for the large outbreaks of neuroinvasive disease (WNND) that have been occurring in Greece since 2010, based on sequence similarities of viral isolates identified between 2010–2012. However, knowledge on the evolution of this strain is scarce because only partial WNV genome sequences are available from Greece. The aim of this study was to get the complete genome sequence of WNV from patients with infection. To this aim, plasma and urine samples collected during the 2012 Greek outbreak were retrospectively investigated. Full WNV genome sequence was obtained from a patient with WNND. The genome had 99.7% sequence identity to Nea Santa, higher than to other related WNV lineage 2 strains, and five amino acid changes apparently not relevant for viral pathogenicity or fitness. In addition, infection by WNV lineage 2 was confirmed in additional nine patients with WNND; in three of them the infection with WNV Nea Santa was demonstrated by sequencing. In conclusion, this study characterized for the first time a WNV full genome from a patient with WNND from Greece, demonstrated the persistence of the Nea Santa strain, and suggested that the virus might have locally evolved.

## 1. Introduction

West Nile virus (WNV) is a mosquito-borne flavivirus that belongs to the Japanese encephalitis virus serocomplex [1]. First isolated in Uganda in 1937, the virus was detected in sporadic cases of disease in Europe in the 1950s, while it was responsible for epidemic outbreaks in Africa and in the Middle East [2]. In 1996, a large outbreak of West Nile neuroinvasive disease (WNND) characterized by a high fatality rate occurred in Romania [3] and, in the following years, in Southern Russia [4,5], while in 1999 the virus was introduced in New York [6], from where it rapidly spread to all North America and caused hundreds of infections each year [7]. The recent years were characterized by a trend towards a more diffuse circulation of the virus and an increase in the number of outbreaks, with human cases of WNND identified in almost all Eastern, Central, and Southern European Countries [8].

In 2010, a major outbreak of WNV human infection with a high mortality rate occurred in Central Macedonia, Northern Greece, with 262 laboratory-confirmed cases of infection, including 197 patients with WNND and 65 patients with West Nile fever (WNF) [9,10,11,12], representing the second largest outbreak recorded in EU after the epidemics in Romania in 1996. Greece was also highly affected by WNV disease in the following years involving several regions with a total of 75 cases of WNND notified in 2011 and 109 cases in 2012 [11,13].

WNV was isolated for the first time in Greece in a mosquito pool trapped in August 2010 in Nea Santa, a village located 30 km northeast of Thessaloniki, where human cases occurred. Full genome sequencing of the virus, defined as “Nea Santa-Greece-2010” [14], revealed that it was lineage 2 and highly similar to the strain that emerged in Hungary in 2004 [15]. This was an unexpected finding, since WNV lineage 2 was considered less pathogenic than lineage 1 and generally not responsible for human disease [16,17].

Subsequently, in Greece, the virus was isolated and/or detected in *Culex pipiens* mosquitoes in 2010, 2011, and 2012 [18,19,20], in a blood sample obtained from a blood donor in August 2010 [21], in a Belgian traveler who visited Kavala, Northern Greece, in 2012 [22], in sentinel chickens and wild birds in 2010, 2011, and 2012 [20,23,24,25,26]. Partial genome sequencing of these WNV isolates demonstrated identity with the Nea Santa-Greece-2010 strain, indicating that the virus had become endemic in Greece. In addition, sequencing and phylogenetic analysis of these isolates showed a high stability of the viral genome with minimum evolution since 2010 [20,24]. However, apart the “Nea Santa-Greece-2010”, the rest WNV sequences from Greece available in GenBank are partial (≤1,878 bp). Furthermore, these fragments are limited to the E, NS3, and NS5 genes, which restrict phylogenetic and genetic analyses on additional genomic regions, *i.e.*, NS1, NS4A, and NS4B, implicated in WNV transmission and/or virulence. In addition, no WNV full genome sequences from human cases in Greece have been reported so far.

To fill the gap on knowledge of WNV full genome sequences from Greece, the aim of this study was to get the complete genomic sequence of WNV from patients with WNV infection. To this aim, plasma and urine samples collected from patients with WNND or WNF during the 2012 outbreak in Greece were retrospectively investigated and the full genome sequence of WNV was obtained from a patient with WNND. Sequencing of the whole viral genome might be useful to monitor WNV circulation and evolution, to detect the emergence of new variants and new genotypes, to identify the presence of mutants not detectable or poorly detected by available molecular diagnostic tests, and to get information potentially useful for vaccine and antiviral drug development.

## 2. Results and Discussion

### 2.1. Detection of WNV Lineage 2 in Patients with Neuroinvasive Disease

Out of 20 patients with a diagnosis of WNV infection for whom plasma and/or urine samples were available for molecular testing, 10 patients with WNND tested positive for WNV RNA. In all cases the presence of WNV lineage 2 was determined by lineage-specific real-time RT-PCR. Among 4 cases, PCR and sequencing of partial regions within the E, NS3, and NS5 genes demonstrated identity with the Nea Santa strain isolated in Greece in 2010 in 3 samples, and the presence of two nucleotide changes in the E gene in 1 sample. One of the two nucleotide changes led to the M483V amino acid mutation compared to the Nea Santa sequence. Since the urine samples from which this variant sequence was obtained had a high viral load, the full viral genome could be sequenced, as detailed in the following section.

### 2.2. Phylogenetic Analysis of a WNV Whole Genome Sequence

**Figure 1:**
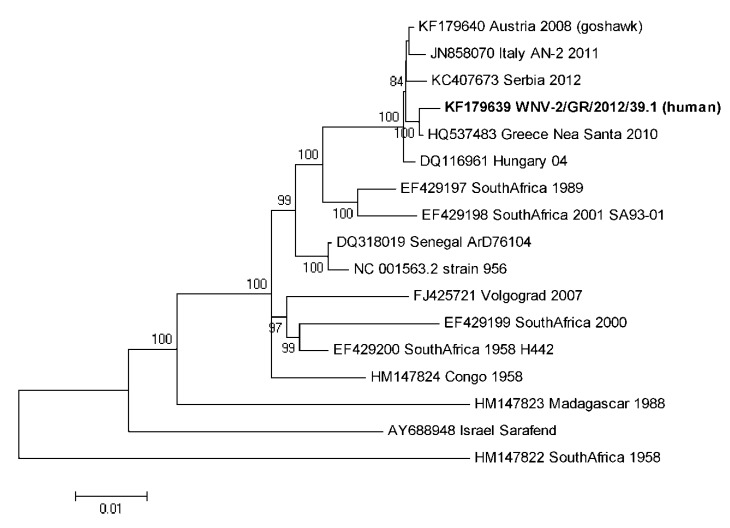
Molecular Phylogenetic analysis of West Nile virus lineage 2 strains by maximum likelihood method. Indicated phylogenetic relationships were inferred with the maximum likelihood method based on the Kimura-2 parameter substitution model and 1,000 bootstrap replicates conducted in MEGA5 [32,33,34]. The analysis involved 17 nucleotide sequences and there were a total of 10,424 positions in the final dataset.

The full genomic WNV sequence was obtained from the WNV-positive urine sample of a 72-year old female patient with WNND in Kavala prefecture. The urine sample was collected at the end of September 2012, 13 days after onset of the illness. This genome sequence represented the first WNV genome fully sequenced from a patient with WNND from Greece. The genome sequence was named WNV-2/GR/2012/39.1 and submitted to GenBank with accession no. KF179639. This strain showed a higher sequence identity to the Nea Santa-Greece-2010 strain than to other related WNV lineage 2 strains within the Hungary/04 cluster [27,28] (Table 1 and Figure 1). The percentage of nucleotide identity with other WNV lineage 2 genomes was as follows: 99.7% identity *vs.* Greece Nea Santa; 99.4% *vs.* Austria 2008; 99.2% *vs.* Italy AN-2 2011; 99.3% *vs.* Hungary 04; and 99.3% *vs.* Serbia. It had a total of 32 nucleotide substitutions and 5 amino acid changes in comparison to the Nea Santa-Greece-2010 strain. Amino acid changes included the R166K and M483V mutations in the E gene, the A146V and the R349K mutations in the NS1 gene, and the V18A mutation in NS2B, as detailed in Table 1. These amino acid changes are conservative and do not seem to affect protein domains predicted to be relevant for WNV pathogenicity or fitness [16], although they were not present in the other closely related WNV lineage 2 isolates (Table 1). In addition, no mutations in 3' UTR and 5' UTR regions of viral genome were demonstrated compared to the Nea Santa-Greece-2010 strain. As detailed in Table 1, both Greek WNV strains have unique amino acid variants that are not present in other isolates of the Hungary/04 cluster, *i.e.*, V119I in NS2B, H249P in NS3, and S14G, T49A, V113M in NS4B. These amino acid changes might have a role in the pathogenicity of this strain, such as the mutation at position 14 of the NS4B gene that has been observed in other WNV lineage 2 pathogenic strains and suggested to be relevant for protein structure [16], and the NS3-T249P substitution identified in several Lineage 1 WNV strains that has been implicated in increased virulence in American crows [29].

Phylogenetic analysis showed closer relatedness of the WNV-2/GR/2012/39.1 to the Nea Santa-Greece-2010 strain than to other fully sequenced WNV lineage 2 strains, within the WNV Hungary/04 cluster of the tree (Figure 1). This analysis of the full viral genome is in agreement with the alignments performed on the NS3 region [20] and indicates that the Greek WNV isolates belong to a distinct evolutionary clade that probably evolved recently [27].

No WNV RNA was detected by nucleic acid testing in 115 cerebrospinal fluids and 9590 blood donor samples collected from seven Greek hospitals during the periods June to October 2006 and 2007, in area involved in the 2010 outbreak [30]. Serological and molecular investigations in wild birds have documented circulation of the virus 8 months prior to the human 2010 outbreak [23]. Furthermore, a study on stored serum samples collected during 2003–2004 from 626 apparently healthy residents of northern Greece showed a seroprevalence of only 0.62%, suggesting that the lineage 2 WNV strain that caused the outbreaks for 3 consecutive years was introduced recently [31].

## 3. Experimental Section

### 3.1. Case Laboratory Investigations

WNV RNA in plasma, urine, and CSF samples was detected by using two different real-time RT‑PCR methods [35], targeting WNV lineage 1 [36] and both WNV lineage 1 and lineage 2 [37], and by a pan-flavivirus nested RT-PCR assay followed by cycle sequencing [38]. Detection of IgM and IgG antibodies against WNV in serum and CSF samples was done by ELISA (WNV IgM capture DxSelect ELISA and IgG DxSelect ELISA kits, Focus Diagnostics, Cypress, CA). To confirm the specificity of antibody response, ELISA-positive samples were further tested by PRNT90, with cutoff 1:10 for positive results, as described [39].

### 3.2. WNV Whole Genome Sequencing

WNV whole genome sequencing was performed on a urine sample with high WNV RNA load collected from a patient with neuroinvasive disease. Total RNA was purified by using the NucliSENS® easyMag® Sytem (bioMérieux SA, Marcy l'Etoile, France). WNV genome was amplified by PCR using a set of 21 primer pairs targeting overlapping sequences of ~600 nucleotides in the WNV genome. Primer sequences are available upon request. Amplicons underwent bi-directional sequencing by using the BigDye® Terminator Sequencing Kit on a 3130 Genetic Analyzer (Life Technologies, Carlsbad, CA, USA). The consensus sequence, obtained by alignment and assembling with the SeqScape v2.5 software (Life Technologies), was submitted to GenBank with accession no. KF179639.

**Table 1:** Amino acid changes in West Nile virus (WNV) lineage 2 full genomes compared to the WNV Greece Nea Santa 2010 genome as reference.

WNV lineage 2 genomes GenBank accession number	Mature peptide amino acid position (polyprotein position)																						
	PrM	E				NS1				NS2A	NS2B		NS3		NS4A	NS4B			NS5				
	16	88	159	166	483	69	146	187	349	1	18	119	244	249	86	14	49	113	25	227	298	446	650
	139	378	449	456	773	860	937	978	1140	1144	1392	1493	1749	1754	2210	2287	2322	2386	2554	2756	2827	2975	3179
**Greece Nea Santa 2010** HQ537483	**A**	**P**	**I**	**R**	**M**	**E**	**A**	**A**	**R**	**Y**	**V**	**I**	**Q**	**P**	**I**	**G**	**A**	**M**	**T**	**G**	**A**	**R**	**T**
**WNV-2/ GR/2012/39** KF179639	.	.	.	K	V	.	V	.	K	.	A	.	.	.	.	.	.	.	.	.	.	.	.
Austria 2008 KF179640	.	.	.	.	.	.	.	.	.	.	.	V	.	H	.	S	T	V	.	.	.	.	.
Hungary 04 DQ116961	.	S	.	.	.	G	.	.	.	H	.	V	.	H	.	S	T	V	A	.	.	.	.
Italy-AN-2 2011 JN858070	V	.	T	.	.	.	.	V	.	.	.	V	.	H	T	S	T	V	.	S	T	H	A
Serbia 2012 KC407673	.	.	.	.	.	.	.	.	.	.	.	V	H	H	.	S	T	V	.	.	.	.	.

Amino acid changes in the WNV lin2 genomes compared to the WNV Nea Santa genome as reference are reported in the table. (.): no amino acid change.

## 4. Conclusions

This study reports the first fully sequenced WNV lineage 2 genome from a patient with WNND from Greece and the second full genome sequence of a WNV from Greece after the Nea Santa-2010 strain. The genome had 99.7% sequence identity to Nea Santa, higher than to other related WNV lineage 2 strains, and five amino acid changes that were apparently not relevant for viral pathogenicity or fitness. In agreement with previous analyses performed on partial sequences of WNV genome [18,19,20,21,22,23,24,25,26], the results of this study indicate that the Greek WNV strain constitutes a distinct evolutionary clade within the Hungary/04 cluster. This strain has become endemic and established a local enzootic cycle in Greece [20,23] where it has spread and acquired some genetic mutations.
